# Comparison between acupotomy and local steroid injection for the management of de Quervain disease: A systematic review protocol

**DOI:** 10.1097/MD.0000000000017765

**Published:** 2019-11-15

**Authors:** Xiaojie Sun, Yifeng Shen, Qiaoyin Zhou, Yan Jia, Zuyun Qiu, Shiliang Li

**Affiliations:** a Department of Acupuncture-Moxibustion, China-Japan Friendship Hospital; b Beijing University of Chinese Medicine, Beijing; c Hospital of Chengdu University of Traditional Chinese Medicine, Chengdu, Sichuan Province; d Fujian University of Traditional Chinese Medicine, Fuzhou, Fujian, People's Republic of China.

**Keywords:** acupotomy, de Quervain disease, de Quervain tenosynovitis, local steroid injection, protocol, systematic review

## Abstract

**Background:**

De Quervain disease (dQD) is a painful condition of the wrist that affects patients’ quality of life and work ability. Acupotomy has been widely used in the treatment of dQD. It has been reported in many articles that acupotomy can improve the clinical symptoms of dQD. However, the efficacy has not been evaluated scientifically and systematically. The aim of this systematic review protocol is to evaluate the efficacy and safety of acupotomy treatment compared with local steroid injection in patients with de Quervain disease.

**Methods:**

Relevant randomized controlled trials will be identified by searching 9 databases (PubMed, EMBASE, Cochrane Library, Chinese literature databases, the Chinese Biomedical Literature Database [CBM], China National Knowledge Infrastructure [CNKI], SinoMed, Technology Journal [VIP], and the Wanfang Database). Randomized controlled trials (RCTs) of Acupotomy for dQD patients will be identified independently by 2 reviewers by searching the databases from inception to October 2018. Clinical effects will be evaluated as the primary outcome. The VAS (visual analog scale) score will be assessed as a secondary outcome. RevMan V.5.3 will be used to perform a fixed effect meta-analysis, and the evidence level will be evaluated by using the Grading of Recommendations Assessment, Development, and Evaluation (GRADE) methods. Continuous outcomes will be presented as the mean differences or standard mean differences, while dichotomous data will be expressed as relative risks.

**Results:**

This study will evaluate the effectiveness and safety of acupotomy in the treatment of de Quervain disease in RCTs with high-quality VAS and RM.

**Conclusion:**

This systematic review will provide evidence to judge whether acupotomy is an effective intervention for patients with de Quervain disease.

**PROSPERO registration number:**

CRD42018108786

## 1 Introduction

De Quervain disease (dQD) is a stenosing tenosynovitis of the first dorsal compartment of the wrist involving the tendons of the abductor pollicis longus and extensor pollicis brevis (abductor pollicis longus [APL], extensor pollicis brevis [EPB]) and presenting as persistent pain and swelling over the radial styloid.^[1–4]^ The pain worsens with abduction of the thumb, grasping action of the hand, and ulnar deviation of the wrist.^[5]^ Additionally, dQD is most frequently diagnosed among women who are 40 to 50 years old.^[6]^ It has commonly been attributed to overfatigue during household duties, but other occupations that include repeated typing or lifting are also known to cause dQD.^[7]^ With a prevalence of 0.5% in men and 1.3% in women among adults of working age in the general population, dQD is relatively common.^[6]^ Female prevalence is 6 to 10 times higher than that in men. Treatment options include oral Non-Steroid Anti-Inflammatory Drugs or corticosteroids, splinting, physical therapy, and surgical decompression.^[8–12]^ Corticosteroid injection is the first choice of treatment for de Quervain disease, and its initial effectiveness rate ranges from 50% to 83%.^[13–16]^ However, 14% to 34.5% of patients may fail treatment.^[16,17]^

The acupotome is a new style of bladed needle that has a flat head and a cylindrical body and evolved from an acupuncture needle.^[18]^ The method of utilizing acupotomes to treat soft tissue injuries and bone hyperplasia has been given the name acupotomy therapy. Acupotomy therapy is considered minimally invasive surgery in traditional Chinese medicine, combining Chinese acupuncture therapy and modern surgical principles.^[19]^ Acupotomy converts open surgery to minimally invasive surgery, thus reducing risk, time, and cost.^[20]^ Acupotomy has been widely used clinically by practitioners of traditional Chinese medicine, orthopedics, and pain departments to treat dQD in China with satisfactory efficacy.^[21–23]^

Acupotomy has been used to treat dQD in China for many years. However, from the perspective of evidence-based medicine, the impact and safety of acupotomy on dQD is still controversial. There is limited evidence in the form of systematic reviews and meta-analysis with regard to acupotomy treatment for de Quervain disease. This study will assess the effectiveness and safety of acupotomy therapy for dQD compared with local steroid injection. To provide evidence for further enhancing the clinical curative effect on patients with dQD, this study will adopt an evidence-based medicine method to analyze and evaluate clinical randomized controlled trials (RCTs) in patients with dQD.

## 2 Methods

### 2.1 Inclusion criteria for study selection

#### 2.1.1 Types of studies

All RCTs on the use of acupotomy therapy in dQD published in any language will be included in this systematic review and meta-analysis. Any study with a sample size <10 will be excluded from this review. Review articles, animal studies, nonclinical studies, and case reports will also be excluded. The research literature will be screened according to the criteria of the review objectives and participants, interventions, comparisons, and outcomes (PICO).

#### 2.1.2 Types of patients

Only studies in which patients have a confirmed clinical diagnosis of dQD will be included. Diagnosis requires radial wrist pain exacerbated by resisted thumb extension. To reflect the condition's widespread nature, no restrictions will be placed upon age, sex, race, or educational status. Fracture and dislocation, muscle injury, bone tuberculosis, bone tumors, and other systematic diseases will be excluded.

#### 2.1.3 Types of interventions

##### 2.1.3.1 Experimental interventions

The review will involve clinical trials that focus on acupotomy treatment (there are no limits on the needle materials, treatment methods, or courses of treatment).

##### 2.1.3.2 Control interventions

Control interventions that include placebo control, steroid injections, drug therapy, block therapy, surgery, no treatment, and acupuncture will be eligible. Evaluations of acupotomy plus another treatment compared with the same treatment alone will also be included. Studies that compare different acupotomy insertions or different forms of acupotomy will be excluded.

#### 2.1.4 Types of outcome measures

##### 2.1.4.1 Primary outcomes

The primary outcome measure of this systematic review will include improvement rates, functional tests, and pain relief. Evaluation will be performed by grip strength and visual analog score (VAS).

##### 2.1.4.2 Secondary outcomes

Roles and Maudsley scores (RM) will be considered as secondary outcomes.

(1) Safety: Safety will be measured by the recurrence rates of dQD, quality of life, and adverse events, such as hemorrhage, serious discomfort, abscess, subcutaneous nodules, and infection.
(2) Acceptance of the measured treatment will be determined by trial exit.

### 2.2 Search methods for the identification of studies

#### 2.2.1 Electronic searches

The following electronic databases will be searched by 2 reviewers from the beginning of each database to October 2018: PubMed, EMBASE, Cochrane Library, Chinese literature databases, the Chinese Biomedical Literature Database (CBM), China National Knowledge Infrastructure (CNKI), SinoMed, China Science and Technology Journal (VIP) and the Wanfang Database. Acupotomy RCTs for dQD will be identified by searching these databases.

#### 2.2.2 Searching other resources

The tables of contents related to dQD and acupotomy, as well as reference lists of the relevant literature and systematic reviews, will also be searched. We will also manually search relevant conference papers and will search Clinical Trials.gov and the WHO International Clinical Trials Registry Platform (ICTRP) for new trials relevant to the topic. The search keywords or combination subject terms will include dQD, acupotomy, small acupotomy, randomized controlled trial, randomized controlled, randomized, controlled, clinical trial, comparative study, prospective study. The accurate Chinese translation of these search terms will be used in the Chinese database. The detailed strategies for searching the PubMed database are presented in Table 1.

**Table 1:**
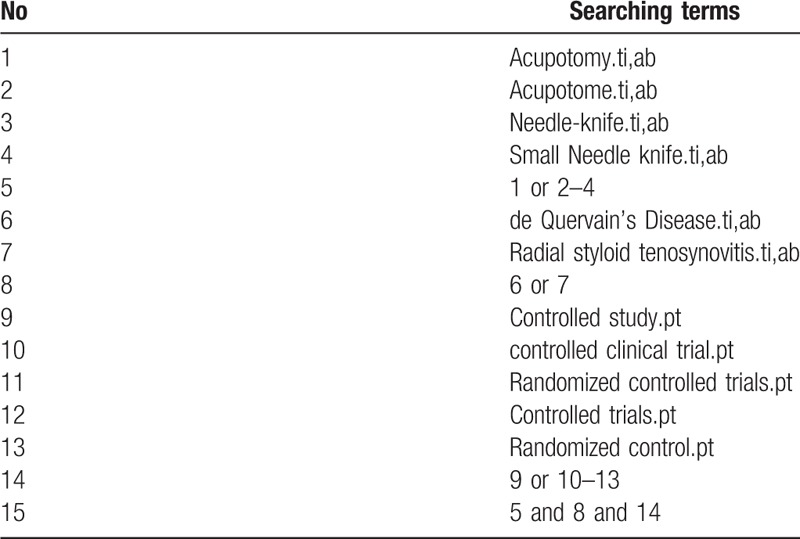
Details of the search strategy for PubMed.

### 2.3 Data collection and analysis

#### 2.3.1 Selection of studies

**Figure 1:**
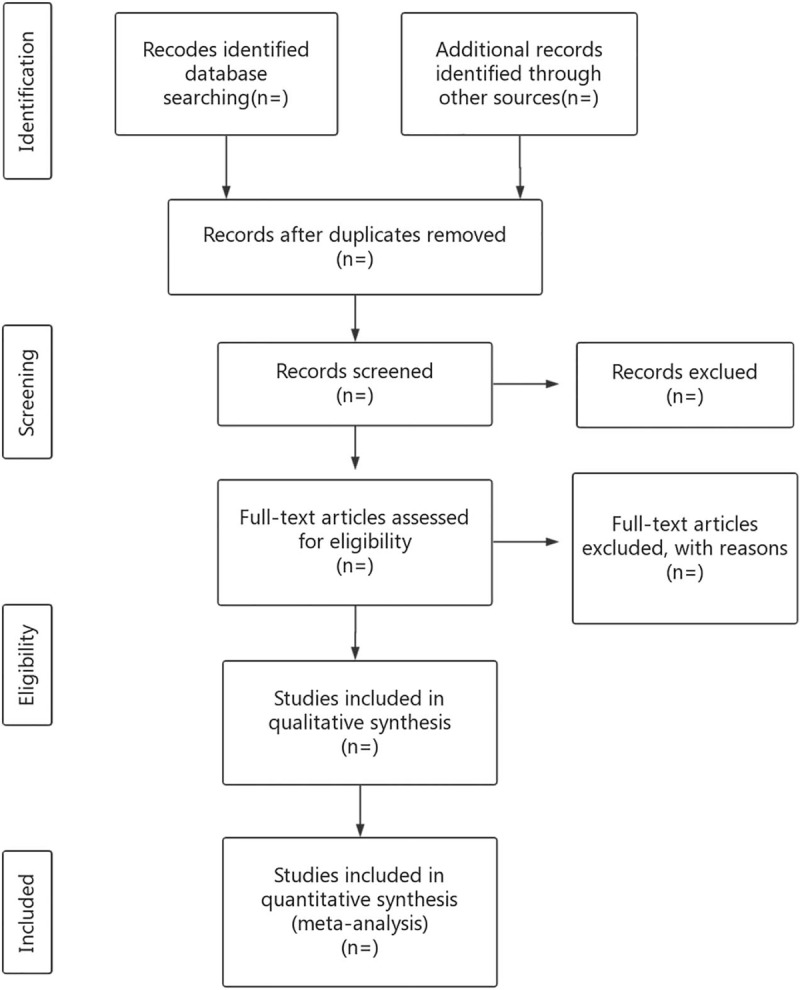
Flow diagram of the study selection process. RCT = randomized controlled trial.

The literature retrieved will be imported by researchers to Endnote library, and duplicate studies will be eliminated. Two reviewers will independently screen articles that are noticeably below standard by reading the title and abstract. Then, the researchers will read the full texts, discuss in the group, and contact the author about the research details to determine final inclusion of the literature (Fig. 1). We will convert the final list of articles into a Microsoft Excel format. Then, the literature search and literature screening will be conducted independently by 2 researchers. Finally, a third independent reviewer will serve as an arbitrator and ultimately make the decision.

#### 2.3.2 Data extraction and management

Data from all the selected eligible articles will be extracted by 2 independent reviewers (XS and YS) into an Excel form. Any discrepancies found will be resolved through discussion and recommendations from the third reviewer (SL). These data collection forms will include the reference ID, author, time of publication, randomization, participant characteristics, country, interventions, blinding, treatment indicators, follow-up, outcome indicators, research results, adverse events, and other detailed information. If necessary, we will contact the trial author for further information.

#### 2.3.3 Assessment of the risk of bias in the included studies

Two independent reviewers will independently use the tools of the Cochrane Collaboration to assess the risk of bias for each included trial. The following 7 aspects will be assessed: random sequence generation; allocation concealment; the blinding method for patients, researchers and outcomes assessors; incomplete outcome data; selective reporting; and other biases as necessary. The risk of bias will be classified as low risk, high risk, and unclear.^[24]^ The results of the evaluation will be cross-checked and resolved through discussion and arbitration by the third reviewer (SL).

#### 2.3.4 Measures for treatment effect

The relative risk (RR) will be used to assess the enumeration data, and the mean difference (MD) will be used to assess the measurement data. The sizes of the effect will be presented for analysis with 95% confidence intervals (95% CIs).

#### 2.3.5 Dealing with missing data

Researchers will contact the corresponding authors to obtain information if there are missing or incomplete data for the primary results. If the missing data are not available, we will perform the analysis based on the available data.

#### 2.3.6 Assessment of heterogeneity

Review Manager (RevMan V.5.3.5 for Windows; the Nordic Cochrane Center, Copenhagen, Denmark) will be used to evaluate the curative effect and publication bias. According to the Cochrane Handbook for Systematic Reviews of Interventions, heterogeneity will be assessed by the *I*^2^ statistic and chi-squared test.^[25]^

We will use the chi-squared test (*α* = 0.1) to analyze the heterogeneity of the research results, and the significance will be determined by the *I*^2^ value. If *I*^2^ ≤50%, the statistical heterogeneity among the trials will be considered negligible, and the size of the effect will be estimated by using a fixed-effects model. *I*^2^ values >50% will be considered evidence of significant heterogeneity among the trials.

#### 2.3.7 Assessment of reporting bias

If there are >10 trials in the study, we will use the visual asymmetry of the funnel plot to assess the reported biases. If funnel plot asymmetry is detected, we will analyze the reasons for this outcome.

#### 2.3.8 Data synthesis

We will adopt RevMan 5.3 software to carry out the meta-analysis. If there is no substantial statistical heterogeneity in the results, the fixed effects model will be used for meta-analysis. If substantial statistical heterogeneity exists, the source of the heterogeneity will be further analyzed. After excluding the effects of significant clinical heterogeneity, the random effects model with 95% CIs will be used for meta-analysis. If there is significant clinical heterogeneity, a subgroup or sensitivity analysis will be performed, or only descriptive statistics will be presented.

#### 2.3.9 Subgroup analysis and heterogeneity studies

If there is obvious heterogeneity in the included trials, we will perform a subgroup analysis based on the severity of dQD and the control intervention.

#### 2.3.10 Sensitivity analysis

If possible, a sensitivity analysis will be performed to verify the robustness of the review conclusions. When sufficient trials are available, we will perform a sensitivity analysis to identify whether the review conclusions are robust according to the following: sample size, the effect of missing data, and methodological quality. In addition, the analysis will be repeated after the exclusion of low methodological quality studies.^[26]^

#### 2.3.11 Grading the quality of evidence

We will assess the quality of evidence by the Grading of Recommendations Assessment, Development and Evaluation (GRADE); quality will be categorized into very low, low, moderate, or high levels.^[27]^

## 3 Discussion

dQD has imposed a burden on individuals, families, and society. Acupotomy treatment for dQD is a minimal surgery with higher acceptability and less pain. Although some trials have shown that acupotomy can effectively reduce the symptoms of dQD, its efficacy has not been evaluated scientifically or systematically. To the best of our knowledge, there are no systematic reviews or meta-analyses of the effectiveness of acupotomy on dQD that have been published. This study will assess the effectiveness and safety of acupotomy therapy for dQD. The purpose of this study is to evaluate the efficacy and safety of acupotomy treatment in patients with dQD. Our systematic review and meta-analysis will be beneficial to patients with dQD, clinicians, and health policy-makers, by providing a deeper understanding of the effectiveness of acupotomy therapy. There are some potential limitations to this review. There may be a risk of heterogeneity in the severity of different types of acupotomy and stenotic tenosynovitis, and the measurements and outcome assessment tools of the included studies may be different.

## Author contributions

SL is the guarantor of the article. The manuscript was drafted by XS and YS. YS and ZQ developed the search strategy. YS and QZ will independently screen the potential studies and extract the data. ZQ and YJ will assess the risk of bias and finish data synthesis. SL will arbitrate any disagreement and ensure that no errors occur during the review. All review authors critically reviewed, revised, and approved the subsequent and final version of the protocol.
